# Amino Acid Transporter SLC6A14 (ATB^0,+^) – A Target in Combined Anti-cancer Therapy

**DOI:** 10.3389/fcell.2020.594464

**Published:** 2020-10-21

**Authors:** Katarzyna A. Nałęcz

**Affiliations:** Laboratory of Transport Through Biomembranes, Nencki Institute of Experimental Biology, Warsaw, Poland

**Keywords:** amino acid transporter, cancer, SLC6A14, estrogen receptor, trafficking, heat shock proteins, AKT 3

## Abstract

Cancer cells are characterized by quick growth and proliferation, demanding constant supply of various nutrients. Several plasma membrane transporters delivering such compounds are upregulated in cancer. Solute carrier family 6 member 14 (SLC6A14), known as amino acid transporter B^0,+^ (ATB^0,+^) transports all amino acids with exception of the acidic ones: aspartate and glutamate. Its malfunctioning is correlated with several pathological states and it is upregulated in solid tumors. The high expression of *SLC6A14* is prognostic and unfavorable in pancreatic cancer, while in breast cancer it is expressed in estrogen receptor positive cells. As many plasma membrane transporters it resides in endoplasmic reticulum (ER) membrane after translation before further trafficking through Golgi to the cell surface. Transporter exit from ER is strictly controlled. The proper folding of SLC6A14 was shown to be controlled from the cytoplasmic side by heat shock proteins, further exit from ER and formation of coatomer II (COPII) coated vesicles depends on specific interaction with COPII cargo-recognizing subunit SEC24C, phosphorylated by kinase AKT. Inhibition of heat shock proteins, known to be upregulated in cancer, directs SLC6A14 to degradation. Targeting proteins regulating SLC6A14 trafficking is proposed as an additional pharmacological treatment of cancer.

## Introduction

All cells need a constant supply of necessary nutrients for their proper functioning, as well as removal of metabolic products. The polar and ionized small molecular weight compounds are capable to cross the lipid bilayer of the plasma membrane due to functioning of transporting proteins - solute carriers (SLC), coded in humans by more than 400 genes. Based on their homology and similarity, the *SLC* genes are divided into 65 families^1^ according to the following convention: *SLCnXm*, where *n* is the number of the family, *X-*the letter indicating a subfamily, *m*-the member of the family. Currently, the traditional names indicating the transporter function and specificity have been replaced by the SLCnXm names also for the proteins.

Among the hallmarks of cancer cells is their ability for quick growth and proliferation (Hanahan and Weinberg, 2011), what relies on energy delivery and on anabolic processes. Several SLCs have been reported to be upregulated in cancer. Among them there is a glucose transporter GLUT1 – SLC2A1 (Yamamoto et al., 1990; Yu et al., 2017), delivering glucose for aerobic glycolysis (so-called Warburg effect), but as well for synthesis of ribose, serine and protein glycosylation. The nucleoside transporters from SLC28 and SLC29 families, found in most, possibly all, cell types, not only deliver substrates for nucleotide synthesis [for review, see Young (2016)], but have become targets for nucleoside-derived drugs used, among others, in the treatment of solid tumors (Pastor-Anglada and Perez-Torras, 2015).

Quickly proliferating cancer cells demand also a supply of amino acids, not only the essential ones. Amino acids are necessary for protein synthesis but also for some other processes, as *de novo* biosynthesis of nucleotides, indispensable in energy metabolism and nucleic acids synthesis, as well as in signal transduction. Glutamine and aspartate are the substrates for *de novo* 6-steps synthesis of pyrimidine nucleotide uridine 5′-monophosphate (UMP) and, after formation of UTP, glutamine is an amino group donor for formation cytidine nucleotide by CTP synthase. A 10-steps buildup of purine nucleotide ring demands glutamine, glycine and aspartate, as carbon donors, beside ATP and N^10^-formyl H_4_-folate (tetrahydrofolate). This leads to formation of inosine 5′-monophoshate, a precursor of GMP and ATP, whose formation demands presence of glutamine and aspartate, respectively. Amino acids are also necessary for tetrahydrofolate metabolism as C1 unit donors: serine and histidine are necessary to form methylene tetrahydrofolate or methenyl tetrahydropholate, respectively. Last, but not least, glutamine, after conversion to glutamic acid is a precursor of non-essential amino acids [for review, see Wise and Thompson (2010)]. Studies with use of ^13^C NMR spectroscopy demonstrated another role of glutamine, namely its high rate metabolism in transformed cells. It has been observed that glutamine contributes to ATP production through conversion to glutamate and α-ketoglutarate (Scalise et al., 2017). Since glutamine uptake in cancer cells exceeds its incorporation to proteins in cancer, its metabolism has been studied in more detail. The tracer studies with [U-^13^C, U-^15^N] L-glutamine showed that it is metabolized in mitochondria and through glutamate and α-ketoglutarate forms fumarate and malate. A part of malate is converted to oxaloacetate and citrate in the TCA reactions. Another part of malate is exported to cytoplasm and is converted to pyruvate by malic enzyme 1 (Murai et al., 2017). This reaction generates NADPH, pyruvate and CO_2_. Citrate exported from mitochondria is a donor of acetyl moiety due to citrate lyase reaction, which produces malate as well [for review, see Wise and Thompson (2010)]. Since both NADPH and citrate formed in the tricarboxylic acid cycle, as a donor of acetyl moiety, are necessary for fatty acid synthesis, glutamine metabolism could lead to augmented membrane synthesis, necessary for cell growth and proliferation. Moreover, it should be added, that another amino acid – serine is a precursor of ethanolamine and choline necessary for the synthesis of phospholipids.

The augmented anabolic processes and the important function of abovementioned amino acids in growth and proliferation result in up-regulation of several amino acid transporters in cancer. They comprise exchangers SLC7A5 (LAT1), SLC7A11 (xCT), SLC1A5 (ASCT2) and a protein catalyzing a net amino acid uptake SLC6A14 (ATB^0,+^) [for review, see Bhutia et al. (2015)].

SLC7A5 (LAT1), highly expressed in many, although not all, cancers (Fuchs and Bode, 2005; Kaira et al., 2008) transports mainly essential amino acids, is specific toward branched-chain- and bulky amino acids and the influx of one substrate is coupled with removal of another amino acid from the cell. SLC1A5, whose historic name ASCT2 comes from alanine, serine and cysteine catalyzes an inward transport of Ala, Val, Met and bidirectional transport of Ser, Thr, Asn, and Gln, while Cys was shown to trigger the efflux reaction, without being transported itself (Scalise et al., 2015). Although the transport is electroneutral, it is coupled with Na^+^ in both directions. It was proposed that the activities of both transporters are coupled: SLC1A5 transports glutamine to the cell in an exchange reaction, while efflux of glutamine is catalyzed by SLC7A5 in an exchange with leucine entering the cell (Scalise et al., 2016). However, the measurements of [^3^H]His transport by LAT1 reconstituted in proteoliposomes showed almost complete inhibition by hydrophobic amino acids Ile, Val, Leu, Cys, Met, while Phe and Ala inhibited His transport to much lower extent. Inhibition by Gln was below 50%, pointing to much lower affinity of SLC7A5 for glutamine (Napolitano et al., 2015). Amino acids, including leucine and glutamine, are necessary for cell growth and proliferation, processes augmented in cancer cells and controlled by mechanistic target of rapamycin (mTOR) regulating cell growth, what leads to protein translation and macrophagy inhibition (Nicklin et al., 2009; Efeyan et al., 2015; Duval et al., 2018). SLC7A11 (xCT) catalyzes uptake of cystine in an exchange with glutamate and, through formation of cysteine, increases the level of glutathione in the cell, protecting the cell from oxidative stress (Lewerenz et al., 2013).

What is interesting, the expression of genes coding these three transporters is regulated by oncogenic transcription factor c-Myc (Hayashi et al., 2012; Bhutia et al., 2015; Wang and Holst, 2015; Grzes et al., 2017; White et al., 2017; Zhao et al., 2019).

### SLC6A14 – A Transporter With Broad Substrate Specificity

*SLC6A14* is coding a plasma membrane transporter called system B^0,+^ (amino acid transporter ATB^0,+^) specific toward neutral (index “0”) and basic (index “+”) amino acids. On the contrary to the amino acid exchangers mentioned above, it transports one molecule of amino acid in a symport with 2 Na^+^ and 1 Cl^–^, using as well the transmembrane potential (Sloan and Mager, 1999).

As presented in Table 1, ATB^0,+^ is a member of SLC6 family of neurotransmitter, osmolyte and amino acid transporters, comprising 4 subfamilies: (i) osmolyte, creatine, GABA transporters, (ii) monoamine neurotransmitter transporters, (iii) amino acid transporters (I), and (iv) amino acid transporters (II) [for review, see Broer and Gether (2012)]. The majority of these transporters were cloned at early 90-ties, especially neurotransmitter transporters. All of them demand Na^+^ and, with the exception of SLC6A15 and SLC6A19, also Cl^–^.

**TABLE 1:** Transporters from SLC6 family.

Transporter	Main substrate(s)	Cotransported ions	References
***GABA, osmolyte, creatine transporters***:			
SLC6A1/GAT1	GABA	2Na^+^, 1C1-	Nelson et al., 1990; Pantanowitz et al., 1993
SLC6A6/TauT	taurine, p-alanine	2Na^+^, 1C1-	Liu et al., 1992a; Ramamoorthy et al., 1994
SLC6A8/CTl/CreaTl	creatine	2Na^+^, 1C1-	Dai et al., 1999; Nash et al., 1994
SLC6A10/CT2/CreaT2	creatine	2Na^+^, 1C1-*	Iyer et al., 1996; Xu et al., 1997
SLC6A11/GAT3	GABA	2Na^+^, 1C1-	Borden et al., 1994; Clark and Amara, 1994; Karakossian et al., 2005; Liu et al., 1993b
SLC6A12/BGT1	betaine/GABA	3Na^+^, 1C1-	Lopez-Corcuera et al., 1992; Yamauchi et al., 1992
SLC6A13/GAT2	GABA	2Na^+^, 1C1-	Borden et al., 1994; Liu et al., 1993b
***Monoamine neurotransmitter transporters***:			
SLC6A2/NET	norepinephrine,		
	dopamine	lNa^+^, 1C1-	Gu et al., 1994; Gu et al., 1996; Pacholczyk et al., 1991
SLC6A3/DAT	dopamine	2Na^+^, 1C1-	Gu et al., 1994; Shimada et al., 1991
SLC6A4/SERT	serotonin	1Na^+^, 1Cl^–^, 1K^+^	Gu et al., 1994; Hoffman et al., 1991
***Amino acid transporters (I):***			
SLC6A5/GlyT2	glycine	3Na^+^, 1C1-	Liu et al., 1993a; Roux and Supplisson, 2000
SLC6A7/PROT	proline	2Na^+^, 1C1-	Fremeau et al., 1992; Galli et al., 1999
SLC6A9/GlyTl	glycine	2Na^+^, 1C1-	Liu et al., 1992b; Roux and Supplisson, 2000
SLC6A14/ATB^0, +^	neutral,		
	cationic amino acids	2Na^+^, 1C1-	Nakanishi et al., 2001; Sloan and Mager, 1999
***Amino acid/nutrient transporters (II):***			
SLC6A15/B°AT2	large, neutral		
	amino acids	lNa^+^	Broer et al., 2006
SLC6A16/NTT5	unknown orphan		
SLC6A17/NTT4	neutral amino acids	lNa^+^	Parra et al., 2008; Zaia and Reimer, 2009
SLC6A18/B°AT3	neutral amino acids	2Na^+^, 1C1-	
SLC6A19/B°AT1	neutral amino acids	lNa^+^	Broer et al., 2004
SLC6A20/SIT1/			
system IMINO	proline, pipecolate,		
	sarcosine	2Na^+^, 1 Cl^–^	Kowalczuk et al., 2005; Takanaga et al., 2005

*GABA, γ-aminobutyric acid; ^∗^, stoichiometry not determined.*

Additionally, the SLC6A4 after translocation of serotonin, Na^+^ and Cl^–^ to the cell, translocates K^+^ outside (Gu et al., 1994).

*SLC6A14* was cloned in 1999 from human mammary gland cDNA (Sloan and Mager, 1999). Its gene is located at chromosome X (Xq23) with the current location 116436606.116461458 (NC_000023.11). It contains 14 exons (each about 100–200 base pairs in length), coding a 642 amino acid protein. Hydrophobicity prediction of this protein suggested 12 putative transmembrane domains with both N- and C-termini localized intracellularly. Such topology, at least with the C-terminus localized at the cytoplasmic side was confirmed with the overexpressed transporter with a C-terminally added tag (3xFLAG), since the transporter was not detected in non-permeabilized cells, while the immunofluorescence signal of anti-FLAG antibody was detected after permeabilization (Samluk et al., 2012). SLC6A14 was not crystallized, anyhow, it has been predicted to have a core structure of the bacterial LeuT transporter (Yamashita et al., 2005). Recently, however, the structural model was proposed using a chimeric model based on the crystal structure of *Drosophila* DAT and the structure of phosphofructokinase from *S. cerevisiae*, as a model for the second extracellular loop (Palazzolo et al., 2019). Using the molecular dynamics simulation and a molecular docking procedure with SLC6A14 substrates, the authors presented a model with 2 binding sites for substrates/inhibitor (S1 and S2) and co-transported ions (Na1 and Na2). They defined several amino acid residues in binding of particular amino acids with Try52, Gly57, Val 128, Ser322 composing an ensemble orienting the substrates. They also proposed Tyr321 at the bottom of S2 site to be involved in gating, while Arg104 and Asp479 define the S1 binding site.

Possibility of several post-translational modifications was predicted from amino acid sequence (Sloan and Mager, 1999).

Phosphorylation by protein kinase C (PKC) has been proven experimentally, moreover, activation of this kinase with phorbol ester augmented level of phosphoserine in ATB^0,+^, a process reversed by PKC inhibitor bis-indolylmaleimide (Samluk et al., 2010, 2012). Phorbol ester treatment resulted as well in a shift of pI of ATB^0,+^ in 2-D electrophoresis (Czeredys et al., 2013). It should be noted that, as demonstrated in experiments using biotinylation of surface proteins, activation of PKC was correlated with an increase of the transporter in the plasma membrane and with the increase of leucine uptake (Samluk et al., 2010, 2012). The observed sensitivity of ATB^0,+^, to phorbol ester treatment suggested an involvement of classical and/or novel isoform of PKC (Newton, 1995) and co-precipitation experiments and immunofluorescence analysis demonstrated an interaction of ATB^0,+^ with the isoform α of PKC (Samluk et al., 2012). Sloan and Mager (1999) predicted also seven possible glycosylation sites on the second putative extracellular loop and one on the third putative extracellular loop. In a model structure of LeuT bacterial transporter glycosylation sites were found at the second extracellular loop, a localization confirmed by crystallization of SERT in which N-acetylglucoseamine electron density were found to be linked to Asn moieties of the second extracellular loop (Coleman et al., 2016). As shown by Kovalchuk et al. (2019), ATB^0,+^ overexpressed in HEK293 cells was sensitive to deglycosylating enzymes: peptide-N^4^-(acetyl-β-glucosaminyl)-asparagine amidase (PNGase F, EC 3.5.1.52) and endoglycosidase (Endo H, EC 3.2.1.96), confirming the presence of glycosylated form of the transporter in the plasma membrane.

*SLC6A14* was shown to be expressed in lung, trachea, salivary gland and also in stomach, mammary gland and in hippocampus (Sloan and Mager, 1999). At the protein level SLC6A14 was detected in colonic epithelium apical membrane (Ahmadi et al., 2018), at the apical membrane of brain capillary endothelial cells forming the blood-brain barrier (Michalec et al., 2014) and in cultured astrocytes (Samluk et al., 2010). It was also detected in rabbit corneal epithelium (Jain-Vakkalagadda et al., 2004).

SLC6A14 with its broad substrate specificity has the highest affinity (K_m_ below 50 μM) for non-polar amino acids Ileu, Leu, Met, Val, and for Ser (Sloan and Mager, 1999). It was as well shown to transport several D-amino acids, such as D-Ser, D-Ala, D-Met, D-Leu and D-Trp (Hatanaka et al., 2002). This can explain the important physiological role of this transporter present in the colon in absorption of amino acids (Ugawa et al., 2001), including the bacteria-derived D-amino acids, in particular D-Ser, known to be an important modulator of N-methyl-D-aspartate (NMDA) receptors in the brain (Matsui et al., 1995). Although D-Ser is synthesized by glial cells (Wolosker et al., 1999), an additional transport of this amino acid by SLC6A14 present in the blood-brain barrier (Michalec et al., 2014) can lead to its increased level in the brain. SLC6A14 was also shown to transport non-proteinogenic amino acids. In the ileum and colon it transports β-alanine (Anderson et al., 2008), a component of the dipeptide carnosine (β-alanine-L-histidine) found in skeletal muscles (Harris et al., 2006). It transports as well L-carnitine (Nakanishi et al., 2001), a compound necessary for transfer of fatty acid acyl moieties to mitochondria, where they undergo β-oxidation [for reviews, see Kerner and Hoppel (2000), Juraszek and Nalecz (2020)]. Although SLC6A14 transports carnitine with a very low affinity in comparison with an ubiquitously expressed high affinity carnitine transporter SLC22A5 (OCTN2), it can partially rescue carnitine supply in case of *SLC22A5* mutations. Such a case was observed in not changed carnitine accumulation in the brain in a mouse strain with functionally defective OCTN2 (Kido et al., 2001). SLC6A14 was also proposed to take over the defective OCTN2 and to mediate transport of butyryl-L carnitine, a compound used for treatment of ulcerative colitis (Srinivas et al., 2007).

Although acidic amino acids are not the substrates of SLC6A14, the β-carboxyl derivatives of aspartate and γ-carboxyl derivatives of glutamate were shown by electrophysiological methods to be transported (Hatanaka et al., 2004). Also the valyl esters of acyclovir and ganciclovir were shown to be transported by overexpressed SLC6A14 (Hatanaka et al., 2004; Umapathy et al., 2004), what indicates a potential role of this transporter as a target for delivery of amino acid-based drugs and prodrugs [for review, see Ganapathy and Ganapathy (2005)]. Moreover, SLC6A14 transports as well the side chain hydroxyl group esters of Ser and Thr (Ganapathy and Ganapathy, 2005) and it was shown to transport zwitterionic or cationic inhibitors of nitric oxide synthase (Hatanaka et al., 2001).

### Physiological Consequences of SLC6A14 Malfunction

As mentioned above, SLC6A14 (ATB^0,+^) is expressed in colon, where it can absorb amino acids from intestinal content, including the D-isomers (Ugawa et al., 2001). The levels of *SLC6A14* mRNA are higher in colonal mucosal specimens from patients with Crohn’s disease, when compared to control samples (Eriksson et al., 2008).

SLC6A14 (ATB^0,+^) is expressed in mammalian blastocysts in species (mouse, rat, human) in which blastocysts undergo the implantation stage with the penetration to uterine epithelium (Schlafke and Enders, 1975), a process regulated by 4-hydroxy-17β-estradiol and β_1_ integrins (Schultz et al., 1997; Paria et al., 1998). It was shown that trophoblast motility was triggered by uptake of leucine through mTOR and polyamine signaling about 20 h before implantation and that activity of ATB^0,+^ was suppressed by uterine environment 12–25 h after estrogen administration (Van Winkle et al., 2006). Therefore, SLC6A14 was proposed to regulate trophoblast motility and to protect blastocysts from immunological rejection and, through supply of amino acids, to influence embryo nutrition and birth weight (Van Winkle et al., 2006). Of note, undernutrition of embryo is correlated with an increased risk of developing obesity. Interestingly, single nucleotide polymorphism (SNP) in the 3′-untranslated region of *SLC6A14* gene was associated with obesity of Finnish population (Suviolahti et al., 2003; Tiwari and Allison, 2003). SNPs in *SLC6A14* were also detected in obese French Caucasians (Durand et al., 2004). It was proposed that this association with obesity resulted from availability of a SLC6A14 substrate – Trp, a precursor of serotonin, which controls appetite (Suviolahti et al., 2003). Another study correlated SNPs in *SLC6A14* gene with susceptibility to male infertility/subfertility in Macedonian/and Slovenian populations (Noveski et al., 2014), a phenomenon proposed to depend as well on availability of Trp and serotonin, associated with testosterone synthesis (Tinajero et al., 1993).

SLC6A14 has been also proposed (Ruffin et al., 2020) to modify the phenotype of cystic fibrosis (CF) – a fatal genetic disorder caused by mutations in the Cystic Fibrosis Transmembrane Conductance Regulator (*CFTR*) gene (Riordan et al., 1989; Rommens et al., 1989). Genome-wide analysis of CF patients from France and North America associated SNPs in *SLC6A14* and *SLC26A9* genes (Sun et al., 2012), the latter one coding the Cl^–/^CO_3_^2–^ exchanger. The authors proposed that variations in these transporters under conditions of loss of CFTR function can be associated with meconium ileus – a severe intestine obstruction at birth, which occurs in 15% of CF patients. Ahmadi et al. (2018) showed in CF-mice model that disruption of *Slc6a14* inhibited by 75% transport of arginine, what worsened fluid secretion in colonic epithelium and affected nitric oxide →cGMP → protein kinase G (PKG) - mediated regulation of CFTR.

### SLC6A14 and Cancer

Expression of *SLC6A14* gene is significantly increased in several human cancer cell lines as well as in patients samples, in particular from solid tumors. The analysis of Cancer Genome Atlas (ACGA) database shows its upregulation in 12 different tumors with the greatest upregulation in pancreatic, cervical and colorectal cancer (Sikder et al., 2017).

RNA sequencing applied to examine expression of genes in the paraffin-embedded tissues of patients with low– and high-grade squamous intraepithelial lesions and compared with the normal cervical epithelium detected *SLC6A14* among differentially expressed and upregulated genes (Royse et al., 2014). Use of commercial array of cDNA from squamous cell carcinoma patients and control normal tissues, as well as the fluorescence studies of cancer specimens demonstrated almost 6-fold upregulation of SLC6A14 at mRNA and protein level in cancer samples (Gupta et al., 2006). Of note, the transporter was observed to co-localize with inducible nitric oxide synthase (iNOS), also upregulated in cervix cancer. Since high levels of nitric oxide (NO) lead to apoptosis and have been associated with protumorigenic effects, SLC6A14 capable of transporting NOS inhibitors could become a therapeutic target through decreasing NO formation. Interestingly, an increase of iNOS was also detected in colorectal cancer samples (Gupta et al., 2005). Semi-quantitative RT-PCR analysis demonstrated about 20-fold increase of ATB^0,+^ mRNA in surgical specimen of patients with colorectal cancer, as well as in metastases in liver and lymph node. Moreover, both transcripts resulting from alternative splicing increased in cancer cells. The increase of SLC6A14 was also detected at protein level what suggests a pathogenic role of the transporter in colorectal cancer (Gupta et al., 2005). Recently, the upregulation of SLC6A14 was reported in most human colon cancer cell lines and in a majority of patient-derived xenografts (Sikder et al., 2020). The deletion of Slc6a14 protected mice from colon cancer, while it’s silencing or blocking with its specific blocker - α-methyltryptophan (α-MT) (Karunakaran et al., 2008) led to a reduced tumor growth and suppressed proliferation due to autophagy and apoptosis. Wnt signaling was reported to be involved in cancer progression, since SLC6A14 expression was shown to be reduced by silencing of β-catenin with shRNA and incubation of cancer cells with Wnt antagonist, while an opposite effect was observed after Wnt agonist treatment and β - catenin overexpression (Sikder et al., 2020). Transcription factor TCF4 (T-cell factor 4) is known to be indispensable for tumor initiation (Hrckulak et al., 2018) and TCF4/β-catenin transcriptional activity mediated by Wnt signaling was proposed to control upregulation of SLC6A14 in colon cancer. This was confirmed by binding of TCF4/β-catenin to promoter of *SLC6A14*, in which four TCF4 binding motifs were found by nucleotide blast (Sikder et al., 2020).

*SLC6A14* was also detected by next generation sequencing in a lymph-node negative subtype of prostate cancer, in one of the most widely represented subtype with the expression of the fusion transcript *TMPRSS2-ERG*, associated with unfavorable survival prognosis (Pudova et al., 2019).

On the contrary to the above-mentioned cancers the transcriptome profiling of biopsies from patients with ulcerative colitis showed that SLC6A14 was among the top 10 downregulated genes (Low et al., 2019).

Karunakaran et al. (2011) showed upregulation of SLC6A14 in estrogen receptor–positive breast cancer tissues and breast cancer cell lines. They found several putative estrogen receptor-binding sites in the *SLC6A14* promoter and confirmed the promoter activity after estradiol treatment with luciferase as reporter. Estradiol treatment increased *SLC6A14* expression in estrogen receptor-positive cell line, while anti-estrogens treatment with tamoxifen reversed this effect (Karunakaran et al., 2011). Treatment of MCF7 - an estrogen receptor-positive cell line with α-MT - a selective blocker of the transporter (Karunakaran et al., 2008) led to amino acid deprivation and induced autophagy, while co-treatment with autophagy inhibitor led to apoptosis and cell death. These phenomena were not detected in SLC6A14 negative cells and the involvement of SLC6A14 was further confirmed by silencing its gene. Moreover, in the mouse xenograft studies administration of *a*-MT reduced tumor volume in case of estrogen receptor-positive cancer cells (Karunakaran et al., 2011). When the *Slc6a14* KO mice were crossed with model mouse lines developing a spontaneous breast cancer, the development of tumor was significantly delayed and its growth was decreased on Slc6a15^–^/^–^ background. Deletion of *Slc6a14* resulted in amino acid deficiency what led to a decrease in mTOR phosphorylation and attenuated expression of genes controlled by HIF1α signaling (Babu et al., 2015). It should be noted, however, that although the majority of breast tumors are estrogen receptor α positive, about 40% of patients acquire resistance to endocrine therapy, affecting either the estrogen receptor itself or the conversion of androgens to estrogens. A global transcription analysis reveals downregulation of *SLC6A14* and an enhanced expression of miR-23b-3p in endocrine therapy resistant breast cancers, what correlates with a prosurvival autophagy and a selective upregulation of *SLC1A2* and results in an increased uptake of Glu and Asp (Bacci et al., 2019). It should be added that miR-23b-3p expression is upregulated by a transcription factor GATA2, reported to promote progression of breast cancer (Wang et al., 2012). Also 4 other miRNA were predicted to regulate SLC6A14 in cancer, their role, however, has to be investigated (Sikder et al., 2017).

Gene expression omnibus database analysis showed 13- to 163-fold increase of *SLC6A14* expression in pancreatic tumors, when compared with normal pancreatic tissue, an increase much higher than the increase of expression of other amino acid transporters: *SLC1A5, SLC7A5, SLC7A11* (Coothankandaswamy et al., 2016). Moreover this upregulation was also confirmed at protein level in a tissue microarray and immunofluorescence quantification (Coothankandaswamy et al., 2016). Many tumors can be detected by imaging techniques such as magnetic resonance imaging or computer tomography, the techniques not sensitive enough to detect the early stages of pancreatic ductal adenocarcinoma, in particular in diffusely infiltrating subgroup of tumors, so finding the diagnostic markers is of high importance. Taking into account the previous reports on functional imaging of sodium iodine symporter in thyroid cancer as well as use of ^18^FDG PET (^18^Fdeoxyglucose positron tomography) imaging to trace the SLC2A1 (GLUT) level known to be upregulated in many cancers, (Penheiter et al., 2015) performed a transcriptomic analysis of in human pancreatic ductal adenocarcinoma samples obtained by laser capture microdissection. The microarray and RNAseq showed 15 SLC transporters being overexpressed when compared with the normal tissue; among them (apart from glucose transporter SLC2A1) lactate transporter SLC16A3 and SLC6A14, which was overexpressed at least 2-fold in cancer patients. This increase in mRNA was further confirmed at protein level and more than 75% of tumor cells were stained with anti-SLC6A14 antibody (Penheiter et al., 2015). Interestingly, the transporter was predominantly detected in the cytoplasm. Based on the data from Protein Atlas^2^ survival prognosis at either low or high *SLC6A14* expression level was found prognostic for pancreatic cancer, being unfavorable at high SLC6A14 expression (Table 2), although the cohorts were relatively small. Another study using an integrated microarray analysis of differentially expressed genes detected 596 upregulated and 540 downregulated genes in pancreatic cancer, when compared with non-tumor control (Yang et al., 2020). *SLC6A14* was found among possible diagnostic and prognostic genes, together with *AHNAK2, CDH3, IFI27, ITGA2, LAMB3*, *and TMPRSS4.* The upregulated genes were annotated to such processes as cell proliferation, apotosis, protein binding and the analysis of enriched genes showed involvement in pancreatic secretion and p53 signaling, MAPK signaling and insulin signaling pathways (Yang et al., 2020).

**TABLE 2:** Analysis of cancer patient survival correlated to *SLC6A14* expression.

Cancer Type	Prognosis	p Value	% 5-year survival		n		FPKM Best cut off	Median Expression
			High	Low	High	Low		
Glioma	Not prognostic	0.0017	0*	11*	33	120	0.01	0
Thyroid	Not prognostic	0.17	92	93	159	342	0.53	0.17
Lung	Not prognostic	0.037	37	50	431	563	7.39	5.56
Colorectal	Not prognostic	0.085	81	57	128	469	5.2	1.71
Head and								
Neck	Not prognostic	0.015	48	38	398	101	0.4	2.46
Stomach	Not prognostic	0.039	35	34	270	84	1.11	4.57
Liver	Not prognostic	0.0074	42	50	93	272	0.02	0
Pancreatic	Prognostic, high expression							
	Unfavorable	0.00097	16	45	107	69	6.08	8.92
Urothelial	Not prognostic	0.16	38	43	126	280	0.65	0.23
Prostate	Not prognostic	0.013	100	96	233	261	1.27	1.12
Testis	Not prognostic	0.021	100	94	77	57	0.02	0.03
Breast	Not prognostic	0.031	83	80	508	567	0.29	0.26
Cervical	Not prognostic	0.036	61	72	178	113	2.17	4.08
Endometrial	Not prognostic	0.0010	81	63	392	144	0.07	0.38
Ovarian	Not prognostic	0.018	35	22	291	82	0.01	0.03
Melanoma	Not prognostic	0.27	36*	26*	28	74	0.56	0.04

The data from Protein Atlas (https://www.proteinatlas.org/ENSG00000268104-SLC6A14/pathology). FPKM, Fragments per Kilobase of exon per Million reads. * 3-year survival.

Another study (Cheng et al., 2019) analyzing differentially expressed genes in pancreatic cancer also detected *SLC6A14* among the top ten upregulated genes and survival analysis of seven of these genes (*TMPRSS4, SERPINB5, SCEL SCL6A14, TMC7, SLC2A1, CENPF*) is associated with patient poor prognosis. The higher expression of these genes was confirmed at protein level with tissue microarray chips and detection with the corresponding antibodies. The retrospective clinical study showed that patients with high level of these genes had significantly shorter survival time. Further experiments with pancreatic cancer cell lines treated with shRNA showed that *SLC6A14* knock-down decreased the invasion of cancer cells, as measured in the transwell assay (Cheng et al., 2019).

This upregulation of *SLC6A14* in pancreatic cancer makes it not only a good candidate as a marker but may make it a drugable target. Experiments performed with pancreatic cancer cells characterized by various *SLC6A14* expression level showed that treatment with α - MT - SLC6A14 blocker, led to amino acid starvation of cells with high SLC6A14. This was not observed in normal pancreatic cell lines. Moreover, blocking of the transporter in the cells with its high expression promoted formation of autophagosomes, suppression of mechanistic target of rapamycin complex 1 (mTORC1) signaling and reduction of migration and invasive properties. α-MT treatment also reduced hypoxia-inducible factor 1α (HIF-1α), usually overexpressed under hypoxic condition of a tumor. Silencing of SLC6A14 with shRNA led also to a decrease in transport of glycine, one of the SLC6A14 substrates. The same authors verified the effect of α-MT treatment in pancreatic cancer cells in mouse xenographs. A marked reduction of tumor growth was observed when α-MT was administered before injection of tumor cells and the treatment was continued, while tumors stopped growing, when α-MT was administered after tumor had grown. A reduction of tumor growth was also observed in xenographs obtained with cancer cells, in which SLC6A14 was knocked-down (Coothankandaswamy et al., 2016). These experiments demonstrated in an elegant way that α-MT can be a pharmacological tool used in tumors with a high SLC6A14 level.

It has to be added that α-MT is a blocker of SLC6A14, while 1-methyltryptophan is a transportable substrate (Karunakaran et al., 2008). 1-Methyltryptophan is used as a pharmacological inhibitor of indoleamine 2,3-dioxygenase (Cady and Sono, 1991), a cytosolic enzyme, whose inhibition activates immune system leading to killing of cancer cells (Hou et al., 2007). So SLC6A14 can be a target in cancer treatment, either by blocking the transporter with α-MT, what results in an arrest of cells at G_1_/G_0_ stage, amino acid deprivation and autophagy, or by affecting tumor-associated immune cells (Karunakaran et al., 2008, 2011).

SLC6A14 can be used as a target for prodrugs, since it accepts esters of hydroxyl group of serine, threonine and tyrosine (Bhutia et al., 2014), as well as drugs conjugated with glutamate and aspartate, such as valacyclovir and valganciclovir (Hatanaka et al., 2004; Umapathy et al., 2004). Beside the abovementioned compounds, the conjugates of acyclovir, ganciclovir irinotecan have been proposed as prodrugs in anti-cancer therapy (Scalise et al., 2020), while butyryl-L-carnitine was proposed for treatment of gut inflammation (Srinivas et al., 2007).

SLC6A14 can be also used as a target for liposomal drug delivery. Aspartate polyoxyethylene stearate conjugate covered liposomes targeting SLC6A14 transporter were shown to increase efficiency of encapsulated docetaxel delivery to human lung cells (Luo et al., 2017). Another study showed that binding of lysine conjugated liposomes to breast cancer MCF7 cells was higher than that of bare liposomes, a process inhibited by α-MT. The further internalization by endocytosis allowed delivery of gemcitabine (chemotherapeutic used in pancreatic cancer treatment) leading to cytotoxicity and, although the transporter is partly degraded, it recovers after some time (Kou et al., 2020). This may lead to novel strategy in nanodelivery of drugs to cancer cells.

It is worth noting that SLC6A14 activity can be detected by a non-invasive method using the cationic amino acid O-2(2-[^18^F]fluoroethyl)methyl-amino)ethyltyrosine in PET (Muller et al., 2014).

### Regulation of SLC6A14

Presence of any plasma membrane transporter at the cell surface, a *sine qua non-condition* of its function, can be regulated at several steps, as transcription of its gene, regulation of translation and trafficking in vesicles to the plasma membrane. Moreover, localization and activity can be regulated by post-translational modifications. As mentioned above, transcription of *SLC6A14* can be regulated by estrogen receptor (Karunakaran et al., 2011) and by TCF4 transcription factor (Sikder et al., 2020). It has to be, however, emphasized, that the prognosis in various types of cancer was based on the mRNA level, what does not fully correlate with the amount of protein, in particular presence in the plasma membrane what is necessary for transporter function.

As any hydrophobic plasma membrane protein, SLC6A14 is inserted to the membrane of endoplasmic reticulum (ER) co-translationally. On the contrary to secreted proteins, it does not have a signal sequence, cleaved later-on^3^. The first transmembrane domain of α-helical structure, formed already within the ribosomal tunnel (Martinez-Gil et al., 2011; Park and Rapoport, 2012; Gogala et al., 2014), is inserted to the ER membrane bilayer by a lateral movement from a proteinaceous channel (Martinez-Gil et al., 2011; Park and Rapoport, 2012), followed by insertion of the other transmembrane helices. The proper folding of any transmembrane protein is strictly controlled. The first steps of glycosylation, so called core-glycosylation, take place in the ER lumen: The initial N-glycan: Glc_3_Man_9_GlcNAc_2_ (Glc – glucose, Man – mannose, GlcNAc – N-acetylglucosamine) is transferred to a nascent polypeptide from dolichol phosphate linked precursor and, after trimming two glucose residues (Moremen et al., 2012) undergoes a quality check, by two lectin chaperons acting in the ER lumen: soluble calretinin and membrane bound calnexin (Lederkremer, 2009; Chevet et al., 2010). As shown by Kovalchuk et al. (2019), SLC6A14 co-localizes with calnexin, moreover, the core-glycosylated species of the transporter, characterized by lower M_r_ than the fully glycosylated one can be detected by Western blot. Inhibition of SLC6A14 exit from ER results in accumulation of core-glycosylated transporter and directs the protein to ER-associated protein degradation (ERAD) (Kovalchuk et al., 2019). The further glycosylation steps take place in Golgi apparatus and lead to fully glycosylated protein (Moremen et al., 2012) which from *trans-*Golgi is trafficked in the vesicles to the plasma membrane.

**FIGURE 1:**
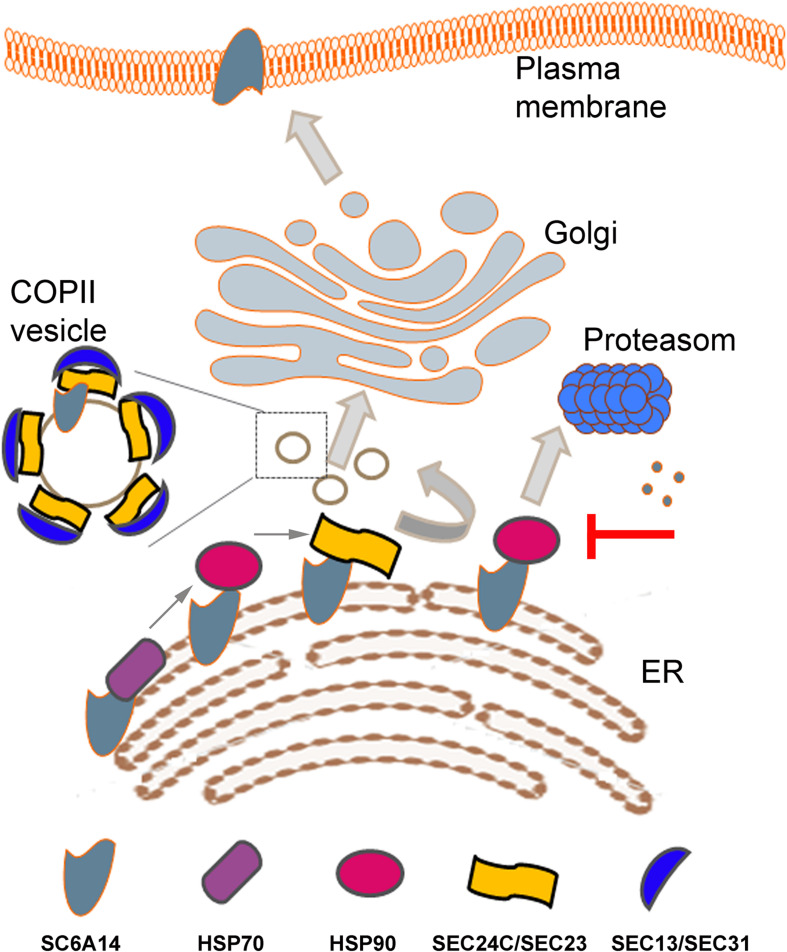
Schematic presentation of SLC6A14 trafficking from endoplasmic reticulum (ER) to plasma membrane. Sequential binding of HSP70, HSP90 and Sec24C in a SEC24/SEC23 dimer is shown in the left bottom part. Inhibition of HSP90 leads to SLC6A14 proteosomal degradation (right, middle part). Coatomer II (COPII) vesicle is shown as a magnified image when vesicles are trafficking between ER and *cis*-Golgi. Details are described in the text.

Apart from the folding control on the luminal side, more and more information is emerging about control of transmembrane proteins at the cytoplasmic side of the ER membrane. As shown previously for adenosine receptor (Keuerleber et al., 2011; Bergmayr et al., 2013) and serotonin transporter (El-Kasaby et al., 2014), the heat shock proteins (HSPs) are involved in the proper folding of the cytoplasmic domain of several plasma membrane proteins. HSPs are highly expressed in various cancers promoting cell survival [for reviews, see Murphy (2013), Lianos et al. (2015)]. HSP70 (HSPA14) and HSP90beta were detected in SLC6A14 proteome and their inhibition with VER155008 and radicicol, respectively led to decreased level of the transporter in the plasma membrane, as assayed by cell surface biotinylation (Rogala-Koziarska et al., 2019; Figure 1). Treatment with HSPs inhibitors attenuated their interaction with SLC6A14 and directed the transporter to endoplasmic reticulum associated degradation (ERAD), a process reversed by proteasome inhibitor bortezomib. Inhibition of HSP70 and HSP90 decreased the amount of SLC6A14 not only after its overexpression, but also in MCF7 estrogen receptor positive breast cancer cell line (Rogala-Koziarska et al., 2019).

The family of HSP70 proteins prevent aggregation of unfolded or misfolded proteins by binding to stretches of exposed hydrophobic residues and the process of unfolding demands both, ATP and co-chaperons [for review, see Murphy (2013)]. Interestingly, several co-chaperons, such as Q8WXX5 (*DNAJC9*) and P31689 (*DNAJA1*) were detected in proteome of SLC6A14 overexpressed in HEK293 cells (Rogala-Koziarska et al., 2019). HSP70 is known to be overexpressed in cancer and its high expression is correlated with increased tumor grade and poor prognosis, in particular in colon cancer, breast cancer, melanoma and bladder cancer (Lianos et al., 2015). Moreover HSP70 overexpression was shown to be a marker of lymph node metastasis in some cancers, including breast cancer (Lazaris et al., 1997). These effects result from HSP70 inhibition of apoptosis, control of cell senescence and autophagy impairment (Murphy, 2013). HSP70 proteins deliver their client protein to HSP90 and it is worth mentioning that the level of HSP90 is elevated in aggressive breast cancers, what makes it a target, at least for diagnostics (Osada et al., 2017). The level of HSP90 is augmented in several cancers (pancreatic, ovarian, breast, lung, endometrial, oropharyngeal, squamous cell and multiple myeloma), what correlates with decreased apoptosis and promotes tumor cell adhesion, motility metastasis and angiogenesis (Wu et al., 2017), therefore it has become a target in cancer treatment [for review, see Wu et al. (2017)]. Out of HSP90 inhibitors, ganetespib seems to be a promising compound as a therapeutic agent (Jhaveri and Modi, 2015). HSP90 forms dimers through its C-terminal domains, while ATPase domain and client protein binding domain are at N-terminus and middle domain, respectively (Wu et al., 2017). We were able to demonstrate a direct interaction between SLC6A14 and both HSPs: HSP90beta and HSP70 (HSPA14). Moreover, we measured the HSP90beta ATPase activity and we detected inhibition of this activity by a peptide QRIIKCCRPASNWGPYLEKH from the C-terminus of SLC6A14, what further confirms the involvement of HSP90beta in the control of SLC6A14 conformation and folding (Rogala-Koziarska et al., 2019). These observations led to a conclusion that inhibition of HSPs can diminish the amount of SLC6A14 in the plasma membrane.

Any plasma membrane protein has to leave ER to reach *cis*-Golgi in the vesicles coated by coatomer II (COPII). Formation of COPII starts from converting Sar1 in a GTP-bound form by Sec12, what recruits a heterodimer Sec24/Sec23 and formation of COPII is completed by binding Sec13/Sec31. Sec24 is a cargo recognizing subunit and in humans there are 4 isomers of this protein, named SEC24A-D [for review, see Miller and Schekman (2013)]. Experiments with over-expressed SLC6A14 showed that this transporter interacts exclusively with the SEC24C isoform and co-expression with SEC24C dominant negative mutant resulted in diminution of the transporter level in the plasma membrane (Kovalchuk et al., 2019). It was proposed that, similarly to other members of SLC6 family (Sucic et al., 2013), motif RIIK within SLC6A14 C-terminal part is responsible for interaction with SEC24C (Rogala-Koziarska et al., 2019). Of note, this motif is within the sequence interacting with HSP90 (Rogala-Koziarska et al., 2019). A relay HSP70/HSP90/SEC24 was proposed as a sequence of binding to A(2A)-adenosine receptor (Keuerleber et al., 2011). These observations lead to the conclusion that inhibition of either HSP70 or HSP90 should prevent binding of SLC6A14 to SEC24C, resulting in directing the transporter to proteasomal degradation (Rogala-Koziarska et al., 2019) and, as a consequence, lowering the amount of SLC6A14 in plasma membrane.

Interestingly, the recombinant Sec24C was shown *in vitro* to be phosphorylated by recombinant kinase Akt (Sharpe et al., 2011), moreover, using the antibodies directed against phosphorylated Akt substrates, the same authors showed phosphorylation of overexpressed Sec24C, when the cells were treated with insulin-growth factor, an up-stream AKT activator. They also detected binding of Sec23 to phosphorylated Sec24C. These observations could suggest a facilitated formation of COPII upon AKT activation.

AKT (protein kinase B) is a serine/threonine protein kinase, known to be activated by an up-stream pathway from activated tyrosine kinase receptors, G-protein-coupled receptors, or integrins, what is followed by activation of phosphatidylinositol 3-kinase (PI3K) and phosphorylation of AKT at T^308^ by phosphoinositide-dependent kinase (PDK-1) [for review, see Nicholson and Anderson (2002)]. This activates AKT and leads to phosphorylation of SIN1, a component of the serine/threonine kinase - mammalian target of rapamycin complex 2 (mTOR2), what is followed by phosphorylation of AKT at S^473^. It should be added that the cell surface receptors are either constitutively active or overexpressed in many human cancers (Harari and Yarden, 2000). Moreover, also the *PKB/AKT* gene was observed to be amplified in several human cancers [see, Nicholson and Anderson (2002)] and AKT pathway is known to be hyperactivated in many types of cancer and dominantly inherited cancer syndromes [for review, see Altomare and Testa (2005)]. It is worth to add that Akt1 deficiency in a mouse model delayed mammary tumor growth and reduced lung metastases (Ju et al., 2007). Active AKT promotes cell survival by phosphorylating proteins regulating apoptotic cascade, just to mention phosphorylation of BAD. AKT is as well involved in the progression of cell cycle, what results in cell proliferation. It phosphorylates complex I of mTOR (mTORC1), thus controlling translation machinery (Broer and Broer, 2017; Thoreen, 2017). Therefore, the cell has a high demand for amino acids, when AKT is active. Although mTORC1 is controlled by a lysosomal amino acid transporter SLC38A9 (Rebsamen et al., 2015; Wang et al., 2015), mTORC2 – a kinase activating AKT (Riaz et al., 2012), conveys the nutrient information from the environment (for review, see Harachi et al., 2018). This suggests that AKT through influence on COPII formation could control transporter exit from ER and its presence in plasma membrane.

## Conclusion and Future Perspectives

Cancer cells are characterized by quick growth and proliferation, demanding a constant supply of nutrients. SLC6A14, being upregulated in many cancers catalyzes a net uptake of all basic and neutral amino acids. In order to fulfill this role, it has to reach the plasma membrane and the first step of trafficking to the cell surface is SLC6A14 exit from ER. This process is promoted by active heat shock proteins HSP70 (HSPA14) and HSP90beta, rescuing the transporter from proteolytic degradation. ER exit of SLC6A14 demands interaction with a component of coatomer II (COPII) – SEC24C, a protein phosphorylated by kinase AKT, known to be hyperactivated in many cancers. Therefore, inhibition of SLC6A14 trafficking before AKT action by inhibition of HSPs seems to be a promising strategy, especially that HSP90 inhibitor – ganetespib has been subjected to phase II clinical trials (Jhaveri and Modi, 2015) in combination with chemotherapeutics used in treatment of several types of cancer. This should lead to a new approach of cancer treatment, for example by inhibiting SLC6A14 with αMT, HSP90 with ganetespib and estrogen receptor with tamoxifen in case of breast cancer.

## Author Contributions

KN was responsible for conception and writing of this manuscript.

## Conflict of Interest

The authors declare that the research was conducted in the absence of any commercial or financial relationships that could be construed as a potential conflict of interest.

## Footnotes

1 http://slc.bioparadigms.org/

2 https://www.proteinatlas.org/ENSG00000268104-SLC6A14/pathology

3 http://sigpep.services.came.sbg.ac.at/results/tempxUDJjT/signalblast.html
